# Snapshot of an intermediate structure

**DOI:** 10.7554/eLife.108402

**Published:** 2025-08-18

**Authors:** Akira Shinohara

**Affiliations:** 1 Institute for Protein Research, The University of Osaka Osaka Japan; 2 Shenzhen University Medical School Shenzhen China

**Keywords:** DNA repair, homologous recombination, RAD51, D-loop, homology search, strand exchange, Human

## Abstract

Cryogenic electron microscopy has been used to determine the detailed structure of an intermediate state called a “D-loop” that forms when strands of DNA are exchanged during homologous recombination.

**Related research article** Joudeh L, Appleby RE, Maman JD, Pellegrini L. 2025. Structural mechanism of strand exchange by the RAD51 filament. *eLife* **14**:RP107114. doi: 10.7554/eLife.107114.

Imagine searching for one object among millions of slightly different ones. This is the challenge during homologous recombination, a process that has multiple roles in genetics. Homologous recombination involves a molecule of single-stranded DNA (ssDNA) ‘invading’ a molecule of double-stranded DNA (dsDNA), and pairing with some or all of the DNA in one of the strands. However, this process – which is called strand exchange (Figure 1A) – can only happen if the invading DNA molecule can find a stretch of DNA with the same sequence in the ‘donor’ dsDNA molecule. The details of this process, which is known as homology search, have been a mystery since Thomas Hunt Morgan and colleagues discovered homologous recombination in the early 20^th^ century.

**Figure 1.**
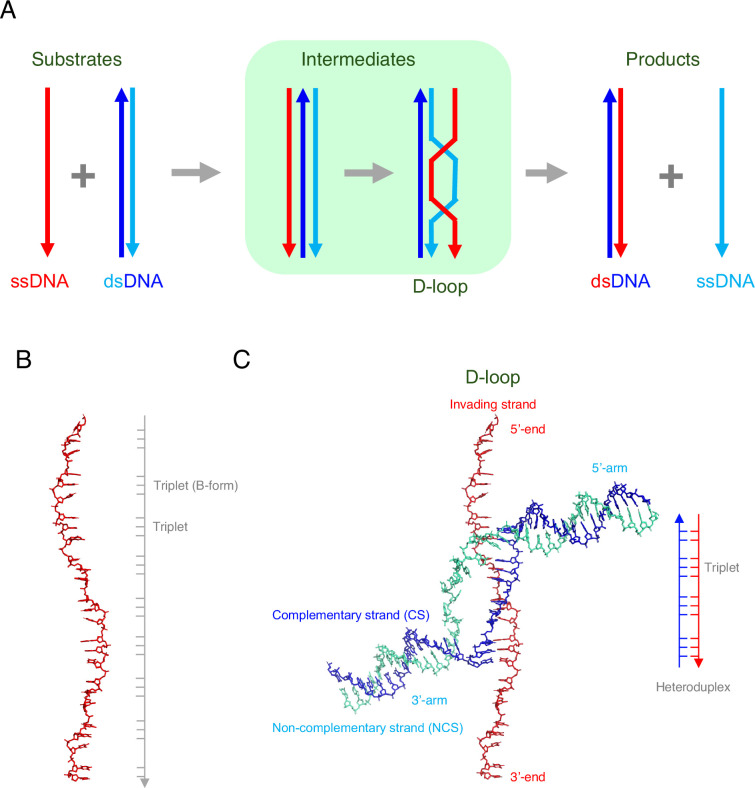
DNA strand exchange and D-loop formation mediated by RAD51. (**A**) A schematic representation of DNA strand exchange. The invading ssDNA (red line) and the donor dsDNA (blue and cyan lines) form an intermediate that contains three strands, followed by an intermediate that contains a structure called a D-loop. The final products of the reaction are: (i) a new dsDNA that comprises the original ssDNA (red) and the complementary strand (blue) from the original dsDNA; (ii) a new ssDNA (cyan), which was previously the non-complementary strand in the original dsDNA. (**B**) Joudeh et al. used an ssDNA with 26 nucleotides in some of their experiments. When bound to RAD51 (not shown), this ssDNA was about 1.5 times longer than the regular (B-form) of DNA. However, the ssDNA was not extended uniformly: rather, most of the extension occurred between the triplets of nucleotides. (**C**) Joudeh et al. used cryogenic electron microscopy to determine the structure of the D-loop structure formed during strand exchange (PDB: 9i62). The D-loop was formed by the invading ssDNA (red) and the complementary strand (blue) of the dsDNA, and contained 11 base pairs (see schematic on the right). The RAD51 molecules are not shown.

During homologous recombination, multiple copies of a recombinase protein bind to the invading ssDNA to form a nucleoprotein filament, with the DNA at the center of this filament being extended by a factor of ~1.5 relative to the regular (B-form) of DNA (Figure 1B). It is this nucleoprotein filament that is responsible homology search. Once the relevant sequence has been found, the ssDNA binds with one of the strands of the dsDNA (the complementary strand) to form a new hybrid molecule of dsDNA; the other (non-complementary) strand is displaced when this happens. A key intermediate stage in this process is the formation of a structure called a D-loop (where D is short for displacement; Figure 1A).

Homologous recombination is conserved across all domains of life, with the recombinase being RecA in prokaryotes, RadA in archaea, and RAD51/DMC1 in eukaryotes. In *E. coli* RecA is a small protein with 352 amino acids and two domains: a central ATPase core domain with two DNA-binding sites, and a C-terminal domain. Human RAD51, which has 339 amino acids, also has two domains: a central ATPase core domain, and an N-terminal domain. However, although the sequence similarity of these two recombinases is only ~30%, they form very similar filament structures when they bind to DNA.

To better understand homology search, it is essential to study the intermediate structures that are formed during the interaction between the nucleoprotein filament and the donor dsDNA. Part of the challenge is that these intermediates – which can contain three strands of DNA – are very short-lived, existing for only a few milliseconds. Now, in eLife, Luay Joudeh, Robert Appleby, Joseph Maman and Luca Pellegrini of Cambridge University report how they have been able to use cryogenic electron microscopy to study some of these intermediate structures (Joudeh et al., 2025).

Joudeh et al. designed ssDNA and dsDNA molecules with sequences that would encourage the formation of D-loops. In one set of experiments the ssDNA molecules contained 32 nucleotides, while the dsDNA molecules had 50, and the nucleoprotein filament contained eight RAD51 molecules. In another set of experiments, there were 26 and 41 nucleotides in the ssDNA and dsDNA, respectively, and nine RAD51 molecules. Moreover, the ends of all the strands were capped with biotin-streptavidin to improve the resolution. In both sets of experiments the matching sequence was ten nucleotides long, as previous research had shown that at least eight matching nucleotides are needed for intermediate structures to form (Hsieh et al., 1992; Lee et al., 2015).

Cryogenic electron microscopy showed that the resulting D-loop contained eleven base pairs (Figure 1C). The displaced (non-complementary) strand is trapped at the edge of a groove in the RAD51 filament, which may prevent the reverse reaction, and six RAD51 molecules are involved in maintaining the stability of the D-loop. Regions of dsDNA not involved in the stabilization of the D-loop are captured by the N-terminal domains of two of the other RAD51 molecules, and are kinked sharply from the filament axis, which is a structural feature not seen before in studies of homologous recombination.

Importantly, the D-loop structure identified in the DNA in human RAD51 is similar to that previously seen in RecA in *E. coli*, except that the regions of dsDNA not involved in the stabilization of the D-loop are captured by the C-terminal domain of RecA (Yang et al., 2020). Thus, both prokaryotic and eukaryotic recombinases are able to form similar D-loop structures, confirming the conserved mechanism of homologous recombination.

This study and others represent a significant advance in our knowledge of RAD51/RecA-mediated homology search and strand exchange (Bell and Kowalczykowski, 2016; Greene, 2016; Prentiss et al., 2015). However, there are still many questions to answer. First, the mechanism by which the ssDNA and the dsDNA find and match with each other remains a mystery. Joudeh et al. propose a model in which the nucleo-protein filament captures the dsDNA, resulting in the formation of an “L2 loop” that locally separates the two strands of DNA, thus allowing interactions between the ssDNA and the separated strands to take place via Watson–Crick hydrogen bonding. However, other models that do not involve strand separation are also possible.

Second, previous studies have shown that: (i) the matching sequences in the ssDNA and the dsDNA must be at least eight nucleotides long; (ii) the filament is stabilized every triplet after the eight base pair strand exchange is established (Hsieh et al., 1992; Lee et al., 2015). The D-loop that Joudeh et al. studied had eleven base pairs (even though the matching sequence was only ten nucleotides long). A challenge for the future is to identify an intermediate with just eight base pairs.

Third, in humans, RAD51 works with other proteins such as BRCA2 (whose mutated form is linked to breast, ovarian and pancreatic cancer), and RAD54 (which is a chromatin remodeler; Ito et al., 2024). How these other proteins influence RAD51-mediated homology search is an important open question.

Fourth, during meiosis, the nucleoprotein filament contains a recombinase called DMC1, rather than RAD51. The DMC1 filament prefers to recombine between homologous chromosomes, whereas the RAD51 filament prefers sister chromatids (Bishop et al., 1992). Understanding the reasons for this difference is another riddle for researchers in the field.

Finally, recent in vivo studies have elegantly shown that RAD51 and RecA filaments sometimes form bundles inside cells (Lesterlin et al., 2014; Liu et al., 2023). Understanding more about this process is a topic for further research.

Since the discovery of RecA-mediated in vitro recombination activities in 1979 (McEntee et al., 1979; Shibata et al., 1979), and RAD51 and DMC1 a decade later (Bishop et al., 1992; Shinohara et al., 1992), the process of homologous recombination has kept researchers searching for an answer to multiple questions.
